# Hepatitis A and E seroprevalence and associated risk factors: a community-based cross-sectional survey in rural Amazonia

**DOI:** 10.1186/1471-2334-14-458

**Published:** 2014-08-23

**Authors:** Claudia Lamarca Vitral, Mônica da Silva-Nunes, Marcelo Alves Pinto, Jaqueline Mendes de Oliveira, Ana Maria Coimbra Gaspar, Rebeca Cristina Costa Pereira, Marcelo Urbano Ferreira

**Affiliations:** Department of Microbiology and Parasitology, Biomedical Institute, Federal Fluminense University, Rua Prof. Hernani Pires de Melo 101, Niterói, RJ 24210-130 Brazil; Laboratory of Technological Development in Virology, Oswaldo Cruz Institute, Oswaldo Cruz Foundation, Rio de Janeiro, RJ Brazil; Department of Parasitology, Institute of Biomedical Sciences, University of São Paulo, Av. Prof. Lineu Prestes 1374, São Paulo, SP 05508-900 Brazil

**Keywords:** Hepatitis A, Hepatitis E, Seroprevalence, Amazon basin

## Abstract

**Background:**

Hepatitis A virus (HAV) and hepatitis E virus (HEV) are both transmitted by the faecal-oral route, and represent common causes of acute hepatitis in developing countries. The endemicity of HAV infection has shifted from high to moderate in Brazil. Human cases of HEV infection seem to be rare, although the virus has been detected in swine livestock and effluents of slaughterhouses. This study was to determine the epidemiology of hepatitis A and E in one of the largest agricultural settlements in the Amazon Basin of Brazil.

**Methods:**

Serum samples collected from 397 individuals aged between 5 and 90 years during a population-based cross-sectional survey were tested for anti-HAV and anti-HEV antibodies. Associated risk factors and spatial clustering of HAV and HEV seropositivity were also analyzed.

**Results:**

The overall rate of HAV seropositivity was 82.9% (95% confidence interval (CI), 79.2-86.6%). Multilevel logistic regression analysis identified increasing age (in years; odds ratio (OR), 1.097; 95% CI, 1.050-1.147; *P* < 0.001) and crowding (OR, 1.603; 95% CI, 1.054-2.440; *P* = 0.028) as significant risk factors for HAV seropositivity. Anti-HEV IgG was detected in 50/388 settlers (12.9%, 95% CI, 9.5-16.2%). Anti-HEV IgM was detected in 7/43 (16.3%) anti-IgG positive samples, and 4 of them had a confirmed result by immunoblot. Increasing age was the only significant determinant of HEV seropositivity (OR, 1.033; 95% CI, 1.016-1.050; *P* < 0.001). No significant spatial clustering of HAV and HEV seropositivity was detected in the area.

**Conclusions:**

Both HAV and HEV are endemic, with differing rates of infection in children and adults in this rural setting of the Brazilian Amazon. Anti-HEV prevalence was considerably higher than those previously reported in Brazil. The detection of HEV- specific IgM antibodies in four asymptomatic individuals is highly suggestive of the circulation of HEV in this rural population.

**Electronic supplementary material:**

The online version of this article (doi:10.1186/1471-2334-14-458) contains supplementary material, which is available to authorized users.

## Background

Despite significant achievements in recent decades to control viral hepatitis worldwide, and a considerable pool of information for prevention, hepatitis A virus (HAV) and hepatitis E virus (HEV) infections remain as matter of a significant public health concern. Both viruses are transmitted primarily by the faecal-oral route, and cause a disease that is indistinguishable without serologic testing. However, HAV and HEV display considerable differences regarding their nature and epidemiology [1]. Immunity to HAV is lifelong, and infection often acquired early in life, while most of the HEV infections occur in late childhood or young adulthood [2]. Moreover, person-to-person transmission of HEV seems to be less frequent and its prevalence, even in endemic areas (7.8% to 45%), not as high as for HAV infection (up to 100% in low-income countries) [3, 4]. Furthermore, HEV is now recognized as a zoonotic virus. Several animal sources of HEV have been identified but swine is considered to be the main reservoir of this virus [5].

HAV and HEV show, both, a worldwide distribution. Their prevalences are very closely related to the level of economic development and access to safe drinking water and sanitation [3, 4]. HEV is associated with sporadic cases and epidemic outbreaks of acute hepatitis ─ both situations related to bad hygiene and sanitary conditions in the regions where such virus is endemic ─ in many Asian and African countries [6]. Nevertheless the global burden of HEV infection is more influenced by sporadically transmitted hepatitis E cases than by epidemics [4]. In industrialized countries, sporadic cases of HEV infection were mainly reported in individuals with a history of traveling to endemic areas. However, indigenous HEV strains ─ genetically different from those circulating in endemic areas ─ have been also reported in Europe, New Zealand, North America, and South America [7–10]. In fact, autochthonous HEV infection has been detected in every country in which it has been sought, usually associated with genotype 3.

In Brazil, the epidemiology of HAV infection has undergone considerable changes. Many studies recently conducted, mainly in urban areas, has shown a consistent decrease of the incidence rates in childhood [11–18]. The country now is considered to have an intermediate level of hepatitis A endemicity [19, 20]. Exposure to this virus has been less frequently investigated in rural populations of this country. Previous studies have shown high seroprevalence rates in riverine populations of the Amazon Basin [21–24], most likely as a result of poor sanitation and lack of safe water supply in these communities. However, a recent study carried out in children living on the periphery of three Brazilian capital cities (Rio de Janeiro, Cuiabá and Manaus) showed that a large number of those under five years old (74.1% - 90%) were susceptible to HAV infection [16]. In contrast, acute cases of HEV seem to be infrequent in Brazil [25, 26]; the first (and single) autochthonous human case of acute HEV infection was reported recently [8]. Among healthy individuals (including blood donors and pregnant women), the anti-HEV IgG seroprevalence ranged from 1.0% to 7.5% [27], with no differences among the geographical regions. Four studies investigated anti-HEV prevalence in the general population of Amazonian communities [23, 28–30], with rates varying from 3.3% to 6.1%.

Here we describe the epidemiology of human hepatitis A and E in one of the largest agricultural settlements in the Amazon Basin of Brazil, the Pedro Peixoto settlement in the state of Acre. We used a multilevel approach to analyze individual and household-level risk factors for the presence of antibodies to HAV and HEV. In addition, we examine the spatial distribution of HAV and HEV seropositive subjects, and discuss the prospects for controlling this infection in this and other similar rural settings.

## Methods

### Study area

**Figure 1:**
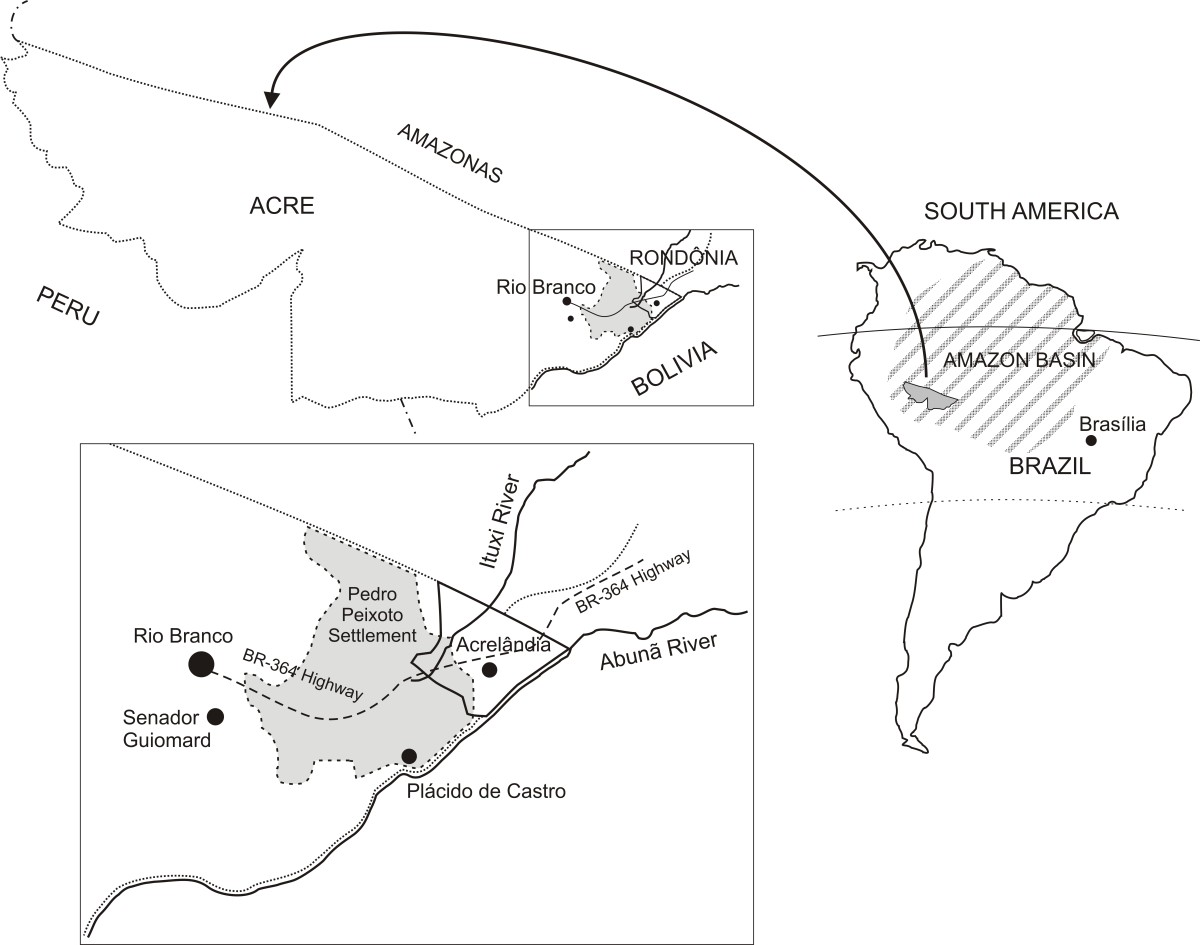
**Location of the municipality of Acrelândia and Settlement Project Director (PAD) Padre Peixoto, known as Ramal do Granada.**

The state of Acre is located in the Western Amazon Basin of Brazil, bordering with Peru, Bolivia and the Brazilian states of Amazonas and Rondônia (Figure 1). The study site, Ramal do Granada (9°41′S-9°49′S, 67°05′W-67°07′W), was a sparsely peopled rubber tapper settlement in the eastern corner of Acre that became part of the Pedro Peixoto Agricultural Settlement Project in 1982. The area is characterized by a humid equatorial climate and receives most rainfall (annual average, 2198.5 mm) between December and March. The mean annual temperature is 24.5°C. Subsistence agriculture and cattle ranching are currently the main economic activities, with coffee, banana and rice as the main cash crops. The high prevalence of intestinal parasites in the study population [31] suggests that prevailing environmental features favour the transmission of enteric pathogens in this community.

### Study population

Recruitment strategies have been described elsewhere [32]. Briefly, all households enumerated during a census performed by our field team in Ramal do Granada were visited between March and April 2004, and 466 dwellers aged less than one year to 90 years of age (98.5% of the 473 permanent residents in the area found at the time of the census) were enrolled. An additional 43 individuals (mostly newcomers to the area) were enrolled between September and October 2004. The 425 study participants aged five years or older who were enrolled either in March-April or September-October 2004 were invited to contribute a 5-mL venous blood sample for serum separation; 397 subjects (93.4% of the eligible; age range, 5–90 years; median, 23 years), living in 118 households, had their sera tested for antibodies to hepatitis A virus and constituted the main population sample analyzed in this survey. In addition, 388 out of 397 subjects whose sera were still available were also examined for antibodies to HEV. The location of all households was determined using a hand-held, 12-chanel global positioning system receiver (eTrexPersonal Navigator, Garmin, Olathe, KS), which gives a positional accuracy within 15 m. A baseline questionnaire was applied to study participants to obtain demographic, clinical and socioeconomic information. The number of years of schooling of the household head, the number of persons per room, and the source of water used for cooking and bathing were recorded. To derive a wealth index, we also obtained information on: (a) the ownership of six household assets (gas stove, couch, bicycle, motor vehicle, and cattle), (b) land tenure (yes or no), (c) the type of housing material (brick walls *vs.* others), and (d) the number of inhabitants per room (≤1 per room or > 1 per room). Principal component analysis was used to define weights for each variable. The first principal component explained 25.6% of the variability and gave greatest weight to ownership of a couch (0.670), a motorized vehicle (car or motorcycle) (0.641) and lower number of inhabitants per room (0.574). Principal component analysis was carried out using the XLSTAT software, version 7.5.2 (Addinsoft, New York, NY). After the standardized variables were weighted, the highest scores were given to the ownership of a brick house (2.262), a sofa set (1.040) and a motor vehicle (0.742). Lowest scores were given to households lacking gas stove (−1.237), with no land tenure (−1.054), with > 1 inhabitant per room (−0.619) and without cattle (−0.614). The scores were summed to a wealth index for each household (range, −4.871 to 5.409).

### Serological assays

Serum samples were screened for HAV and HEV specific IgG antibodies by using the commercial enzyme linked immunosorbent assays (ELISA): bioELISA HAV IgG and bioELISA HEV IgG (Biokit, Spain) according to manufacturer’s instructions. Reactive samples in the IgG hepatitis E assay were repeatedly tested and considered positive only if reactive in duplicate. The anti-HEV IgG reactive samples were also tested for anti-HEV IgM by using two immunoassays, bioELISA HEV IgM (Biokit, Spain) and recomWell HEV IgM (Mikrogen, Germany). The reactivity of samples in HEV ELISAs was confirmed by immunoblot assay (IB) recomLine HEV IgM/IgG (Mikrogen, Germany). Both Mikrogen assays, ELISA and IB, are based on genotypes 1 and 3, while the bioELISA HEV IgM/IgG contains type- common recombinant HEV antigens derived from Burmese and Mexican strains (genotypes 1 and 2 viruses).

### Data analysis

A database was created with SPSS 13.0 (SPSS Inc, Chicago, IL). Prevalence rates are given with exact binomial 95% confidence intervals (95% CI) and compared with χ^2^tests or χ^2^tests for trend; unadjusted odds ratios were also calculated for potential risk factors. Multiple logistic regression models with stepwise backward deletion were built to describe independent associations between potential risk factors (independent variables) and HAV and HEV seropositivity. Variables associated with *P* values <0.20 in unadjusted analysis were included into logistic regression models. Because the data have a nested structure, where individuals are nested within households, the assumption of independence of observations underlying standard logistic regression analysis is violated. We therefore used two-level logistic models with individual-level covariates (age, gender, and history of previous hepatitis) and household-level risk covariates (education of the household head, wealth index, and source of water for cooking and bathing). The HML software package (version 6.03, Scientific Software International, Lincolnwood, IL) was used for multilevel analysis. Only variables associated with statistical significance at the 10% level were maintained in the final model.

The Kulldorff spatial scan statistics was used to test whether HAV and HEV seropositivity was randomly distributed within the study area and, if not, to identify significant spatial clusters (Kulldorff and Nagarwalla, 1995). Analysis was made using the Bernoulli model implemented in the version 5.1 of the SaTScan software (available at: http://www.satscan.org), which creates and moves circular windows systematically throughout the geographic space to identify significant clusters of infections. The windows are centered on each household; the largest possible cluster would encompass 30% of the households. For each location and size of the scanning window, SaTScan performs a likelihood ratio test to evaluate whether or not HAV and HEV seropositivity is significantly more prevalent (high-prevalence clusters) or less prevalent (low-prevalence clusters) within than outside that given circular window. *P* values were determined by 10,000 Monte Carlo replications of the data set; a level of significance of 5% was adopted.

### Ethical considerations

Approval of the study protocol was obtained from the Ethical Review Board of the Institute of Biomedical Sciences of the University of São Paulo, Brazil (318/2002). The study was performed in compliance with relevant laws and institutional guidelines and in accordance with the ethical standards of the Declaration of Helsinki. Written informed consent was obtained from all study participants or their parents/guardians.

## Results

### Prevalence of hepatitis A antibodies and associated risk factors

HAV antibodies were detected in 309 subjects (median, 27 years; interquartile range, 16–40 years), with an overall seroprevalence rate of 82.9% (95% CI, 79.2-86.6%). The seroprevalence rate was substantially higher among subjects older than 30 years of age (96.0%) than in preschool and schoolchildren aged 5–14 years (59.7%), consistent with most of the enterically transmitted diseases occurring in early childhood. The seropositivity rate was slightly higher in females (84.7%) than in males (81.1%), a difference without statistical significance in unadjusted analysis (Table 1). No significant association was found between HAV seropositivity and a past history of hepatitis (Table 1). No household-level variable was significantly associated with the presence of HAV antibodies in unadjusted analysis (Table 1). After adjustment for confounding covariates by using two-level logistic regression analysis, only increasing age, gender and crowding emerged as significant (*P* < 0.05) independent predictors of HAV seropositivity (Table 2). The Kulldorf spatial scan statistic revealed no significant high or low HAV seroprevalence cluster in Granada.

**Table 1:** **Prevalence of HAV antibodies according to individual and household-level risk factors**

Variable	No. of subjects^a^	HAV antibody prevalence (%)	Odds ratio (95% CI)	*P*
Age (years)				
5-10	69	46.4	1.00	<0.0001^b^
11-20	111	80.2	4.68 (2.28-9.61)	
21-30	67	95.5	24.67 (6.84-131.24)	
31-50	103	97.1	38.54 (10.82-203.18)	
>50	47	93.6	16.96 (4.63-91.28)	
Sex				
Female	190	84.7	1.29 (0.74-2.26)	0.417
Male	207	81.1	1.00	
Past history of hepatitis				
No	345	81.4	1.00	0.082
Yes	52	93.2	2.73 (0.95-10.79)	
Education of household head (years of schooling)				
0	81	87.6	2.12 (0.76-6.01)	0.418^b^
1-4	186	77.4	1.20 (0.52-2.63)	
5-8	84	88.1	2.22 (0.80-6.26)	
>8	52	76.9	1.00	
Wealth index (quartiles)^c^				
1 (poorest)	112	83.0	1.06 (0.47-2.39)	0.973^b^
2	101	82.2	1.00 (0.44-2.28)	
3	100	84.0	1.14 (0.49-2.66)	
4 (least poor)	84	82.1	1.00	
Water source				
Well	375	82.4	1.00	0.462
River/stream	22	90.9	2.14 (0.50-19.27)	
Drinking water filtrated or chlorinated				
Yes	308	83.4	1.00	0.523
No	73	79.4	0.77 (0.39-1.58)	
Crowding (number of inhabitants/room)				
<1	168	86.9	1.00	0.930^b^
1-1.9	187	77.5	0.52 (0.28-0.94)	
2-3	18	77.8	0.53 (0.15-2.41)	
>3	24	100	Not calculable	

Ramal do Granada, Brazil, 2004.

^a^Number of individuals differ for some variables, because of missing values.

^b^ *P*values forχ^2^ tests for linear trend; all other *P* values are for standard χ^2^ tests.

^c^Wealth index derived from information on household assets and other socioeconomic data; see the “Subjects, Methods” section.

95% CI, 95% confidence interval.

**Table 2:** **Results of the final multilevel logistic regression model including variables putatively associated with HAV antibodies**

Variable	Odds ratio	(95% CI)	*P*
Age (in years, continuous variable)	1.097	(1.050-1.147)	<0.0001
Sex (male *vs.* female)	0.594	(0.381-0.929)	0.022
Crowding (number of inhabitants/room)	1.603	(1.054-2.440)	0.028

Ramal do Granada, Brazil, 2004.

95% CI, 95% confidence interval.

### Prevalence of hepatitis E antibodies and associated risk factors

HEV antibodies were detected in 50 subjects, with a median age higher than that of HAV-seropositive subjects (median, 31 years; interquartile range, 23–45 years). The overall HEV positivity rate was 12.9% (95% CI, 9.5-16.2%), with the highest seroprevalence (19.4%) in young adults (21–30 years of age) (Table 3). The only household-level variable significantly associated with the presence of HEV antibodies in unadjusted analysis was the education of the household head (Table 3). After adjustment for confounding covariates by using two-level logistic regression analysis, only young age remained as a significant independent predictor of HEV seropositivity, with an OR of 1.033 (95% CI, 1.016-1.050; *P* < 0.001), indicating that each additional year of age increased the odds of having HEV antibodies by 3.3%. The Kulldorf spatial scan statistic revealed no significant high or low HEV seroprevalence cluster in Granada.

**Table 3:** **Prevalence of HEV antibodies according to individual and household-level risk factors**

Variable	No. of subjects^a^	HEV antibody prevalence (%)	Odds ratio (95% CI)	*P*
Age (years)				<0.008^b^
5-10	66	9.1	1.00	
11-20	107	4.7	0.49 (0.11-2.03)	
21-30	67	19.4	2.41 (0.78-8.23)	
31-50	101	17.8	2.17 (0.76-7.05)	
>50	47	17.0	2.05 (0.57-7.72)	
Sex				
Female	184	14.1	1.23 (0.65-2.34)	0.290
Male	204	11.8	1.00	
Past history of hepatitis				
No	337	13.3	1.00	0.631
Yes	52	9.8	0.71 (0.21-1.91)	
Education of household head (years of schooling)				0.046^b^
0	81	18.5	2.73 (0.80-11.92)	
1-4	174	13.2	1.83 (0.58-7.61)	
5-8	81	9.9	1.32 (0.33-6.29)	
>8	52	7.7	1.00	
Wealth index (quartiles)^c^				
1 (poorest)	109	12.8	1.60 (0.57-4.92)	0.497^b^
2	98	14.3	1.81 (0.64-5.57)	
3	98	15.3	1.96 (0.70-5.99)	
4 (least poor)	83	8.4	1.00	
Water source				
Well	366	13.4	1.00	0.382
River/stream	22	4.5	0.31 (0.01-2.01)	
Drinking water filtrated or chlorinated				
Yes	302	13.2	1.00	0.961
No	72	13.9	1.06 (0.45-2.30)	
Crowding (number of inhabitants/room)				
<1	161	13.7	1.00	0.673^b^
1-1.9	181	12.1	0.87 (0.44-1.73)	
2-3	22	18.2	1.40 (0.32-4.83)	
>3	24	8.3	0.57 (0.06-2.63)	

Ramal do Granada, Brazil, 2004.

^a^Number of individuals differ for some variables, because of missing values.

^b^ *P*values forχ^2^ tests for linear trend; all other *P* values are for standard χ^2^ tests.

^c^Wealth index derived from information on household assets and other socioeconomic data; see the “Subjects, Methods” section.

95% CI, 95% confidence interval.

Of the 50 anti-HEV IgG positive samples, 43 were also tested for detection of anti-HEV IgM, by ELISA. HEV IgM antibodies were detected in 7 (16.3%) individuals, from which 6 samples were still available for additional testing with the recomLine HEV IgM/IgG immunoblot (IB) assay. Four of these samples tested positive in the recomLine HEV IgM/IgG. The two samples that tested negative by IB showed low (1,3 and 1,6) optical density/cutoff (OD/CO) ratios. On the other hand, the four IB positive samples had higher ratios (between 3 and 6). These four IgM anti-HEV/IB positive individuals (aged 10, 25, 28 and 34 years, and living in the study area since 5 to 19 years) were asymptomatic, and also positive for HAV IgG antibodies. Regarding the two individuals whose HEV IgM reactivity was not confirmed by IB, one (Male, 31 years) had malaria 30 days before blood collection, and positive serology for Mayaro virus at the time of blood collection; from the other, data are not available. One presumptive anti-HEV IgM positive sample (according to the bioELISA HEV IgM manufacturer’s instructions), which showed very low (1,1) OD/CO ratio, was from a 38 years old pregnant woman, with vivax malaria at the time of blood collection.

## Discussion

This is the first community-based survey that compares, in the same population in Brazil, prevalence and risk factors for HAV and HEV infection. Poor sanitation and lack of access to safe water usually lead to enteric infections in early childhood, consistent with the HAV age-prevalence data described here for the Granada community (Table 1), and also observed in low socioeconomic status groups in Brazil [23, 28, 33, 34], and other countries in Latin America [3, 35]. Since we have excluded children below 5 years of age from the study population (because many children’s parents or guardian refused to consent a venous blood sample), we were unable to estimate the force of HAV infection over the first years of life in this highly-endemicity area.

Regarding HEV infection, it tends to affect all age groups in Granada, as observed for other Brazilian communities [23, 28–30, 33, 36–38], (Table 3). However, the highest seroprevalence was found in young adults, in agreement with other HEV seroprevalence studies [2, 39]. Of interest, the rate of anti-HEV IgG seropositivity observed (12.9%) was substantially higher in comparison to those found in several subsets of healthy individuals across the Amazon Basin. Previous studies reported rates of 6% in gold miners [29], 3.3% in a general population of the Southern Amazon basin [30], 4.5% in children aged 2 to 9 years old from an Amazonian municipality in Mato Grosso State [28], and 4% in riverine communities from the Western Amazon basin [23]. Anti-HEV IgG rates reported from other Brazilian regions, as well as from other Latin American countries including either urban or rural population, ranged from 1% to 10% [27].

It should be mentioned that the performance of diagnostic assays for hepatitis E is still a matter of concern, and may compromise comparison among different HEV seroprevalence studies [40]. According to Khudyakov and Kamili [41], it seems that the observed poor concordance among assays for detection of IgG anti-HEV may be explained by variation in sensitivity rather than specificity of these assays. However, doubts about the specificity of these assays also emerged in the last years [40], which could means that the prevalence record could actually be higher than the real. As a matter of fact, the specificity of the available assays can vary widely [42]. For instances, in blood donors from the United Kingdom anti-HEV IgG was detected in 3.6% and 16.2% by using different commercial assays [43]. Similarly, in a setting of patients with acute HEV infection (with HEV RNA detectable), 44% of the samples were reactive with one assay and 98% with the other [42]. It is a general concern that efforts should be done in order to establish a consensus about the most suitable technologie for performing serosurveys. The commercially available assays exhibit varying diagnostic properties of antigens [41]. Besides, the duration of antibody response to different HEV epitopes was shown to vary widely [44]. In the present study, one sample (which showed a high seropositivity of both, bioELISA and recomWELL IgM, and also, IgG bioELISA) had IgM positive (reactive for O2 and O3 antigens) but IgG negative results by IB. The opposite was observed for another sample that were IgG positive (reactive for O2, but non-reactive for O3 antigen), but IgM negative by IB. This sample, however, had high ratios of IgM seropositivity detected by both, commercial (Bioelisa and Recomwell) immunoassays. Such discrepant results may reflect the timing of sampling as also described by others [45].

In general, prevalence of HEV IgG antibodies does not necessarily reflects the disease prevalence, especially in regions of low endemicity. However, confirmed reactivity of anti-HEV IgM is considered diagnostic for acute infection [40]. In the present study, the detection of HEV- specific IgM antibodies (confirmed by IB) in 9.3% (4/43) of the samples obtained from a setting of healthy subjects is highly suggestive of the circulation of HEV in the actual population.

Among the risk factors considered in determining the patterns of prevalence in developing countries, age and crowding emerged as significant contributors to HAV seropositivity in Granada. In contrast with previous studies conducted in large and heterogeneous Brazilian cities [46, 47], however, neither socioeconomic variables such as wealth index and educational level of the household head, nor environmental variables such as access to safe water, were significantly associated with HAV seropositivity in this relatively homogeneous rural community. A gender-related difference in HAV seropositivity rate was observed, although it has been rarely reported elsewhere. For example, females were found to be more frequently seropositive for HAV than males in a large population-based survey in São Paulo, the largest city in Brazil, but the difference in seroprevalence rates (67.7% vs.65.5%) did not reach statistical significance [48].

No environmental risk factor was significantly associated with HEV seroprevalence in Granada. Interestingly, although the local environmental conditions theoretically favour the transmission of enteric pathogens (and, in fact, intestinal parasites are rather frequent in this population [31]), our data indicates that in Granada HEV infection is acquired later than HAV. And, although the mode of transmission of HEV was not clear, it seems that the pattern of HEV infection in this rural Amazonian community is rather related to that observed for HEV genotype 3 in developed countries, where the majority of cases, when the source of infection can be determined, occurs due to consumption of contaminated shellfish, undercooked pork or wild game and direct exposure to pigs. HEV genotypes 1 and 2 (HEV1, HEV2) are exclusive of human beings, restricted to particular geographical areas (HEV1, Southern and Central Asia, the Far East, and the Caribbean; HEV1 and-2, Africa), and spread often among the population as waterborne, open epidemic outbreaks. Moreover, looking at the patterns of acquisition of anti-HEV with age among the population from regions endemic for HEV1, it can be observed that anti-HEV is acquired earliest in life in regions endemic for HEV1 in comparison with regions endemic for HEV3 [40]. India, Malaysia and Southern China displayed anti-HEV rates among children up to 20-50% [49–51], although similar or even lower rates than the one reported here (9%) had been found in India (0.6 – 8.9%) [52]. In Brazil, HEV 3 has been the only genotype detected, either in acute hepatitis patients or in pigs [8, 51, 53, 54]. Evidences of HEV circulation in the Brazilian Amazon and neighboring countries have been also reported. HEV genotype 3 has been detected in faecal samples collected from pigs from different areas of the Pará state, located in Eastern Brazilian Amazon [55], and in human and swine faecal samples from two rural communities in southeastern Bolivia [56]. However, the involvement of HEV genotype 1 could not be ruled-out totally, since it has been already found in infections acquired locally at Venezuela [57], Uruguay [58], and Cuba [59]. Unfortunately, the study might have been underpowered in detecting significant associations with specific risk factors, since data about contact with domestic or wild animal were not systematically collected. Likewise, the HEV genotype circulating in Granada could not be identified because environmental and stool samples were not available from the studied population.

The prevalence of anti-HAV was much higher than that observed for anti-HEV, which is consonant with the profile reported from endemic areas for both viruses. It has been noted that, even in regions where both viruses are endemic, the prevalence of HEV is considerable lower than the observed for HAV [60]. A cross sectional study in the general population of Tehran, Iran, showed prevalence rates of HAV and HEV IgG antibodies of 90% and 9,3%, respectively [61]; in Aden, Yemen, Bawazir et al. [62] reported anti-HAV and anti-HEV prevalence rates of 86.6% and 10.7%, respectively, among individuals attending primary health care facilities. Rates of 94.1% and 7.3% for anti-HAV and anti-HEV were found by Bartoloni et al. [63] in the population of two rural areas in south-eastern Bolivia. To which extent these age-structured seroprevalence data are affected by a decline in HEV antibody titers over time which could lead to an underestimation of the frequency of HEV exposure as previously evidenced remains to be investigated.

## Conclusion

In conclusion, both HAV and HEV infections seemed to be endemic in this Amazonian community. Even though the prevalence rate of HEV had been much lower than the rate observed for HAV, it was considerably higher than anti-HEV rates previously reported for several population groups in Brazil. Moreover, the detection of HEV- specific IgM antibodies in four asymptomatic individuals is highly suggestive of the circulation of HEV in this rural population. Further studies are required to identify specific risk factors that would be involved in HEV circulation within this tropical Amazonian setting.

## Authors’ original submitted files for images

Below are the links to the authors’ original submitted files for images. Authors’ original file for figure 1
