# Supplementary figures and images for: Hepatitis A and E seroprevalence and associated risk factors: a community-based cross-sectional survey in rural Amazonia
Source: BMC Infect Dis. 2014 Aug 23;14:458. doi: 10.1186/1471-2334-14-458 (PMC4152586; doi:10.1186/1471-2334-14-458)

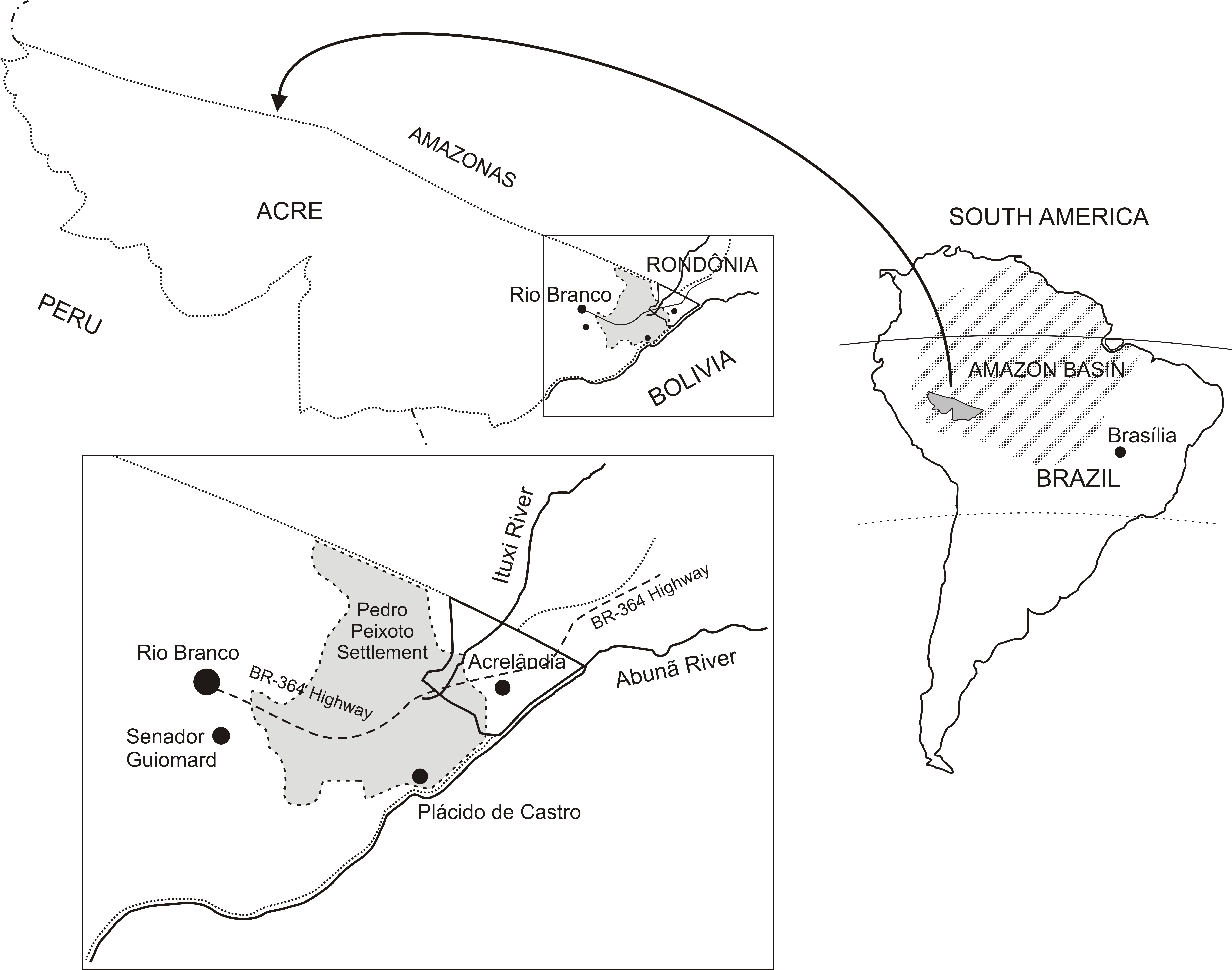

Supplement: Supplementary file 1 — Authors’ original file for figure 1 [file 12879_2014_3754_MOESM1_ESM.jpeg]
